# Paediatric acute myeloid leukaemia with the t(7;12)(q36;p13) rearrangement: a review of the biological and clinical management aspects

**DOI:** 10.1186/s40364-015-0041-4

**Published:** 2015-10-05

**Authors:** Sabrina Tosi, Yasser Mostafa Kamel, Temitayo Owoka, Concetta Federico, Tony H. Truong, Salvatore Saccone

**Affiliations:** Leukaemia and Chromosome Research Laboratory, Division of Biosciences, Brunel University London, Middlesex, UB8 3PH UK; Dipartimento di Scienze Biologiche, Geologiche e Ambientali, Sezione di Biologia Animale, University of Catania, Catania, Italy; Division of Pediatric Oncology, Blood and Marrow Transplant, Alberta Children’s Hospital, University of Calgary, Calgary, Canada

**Keywords:** Acute myeloid leukaemia, Paediatric leukaemia, t(7;12) translocation, Chromosomal abnormalities, *HLXB9* gene, Clinical outcome

## Abstract

The presence of chromosomal abnormalities is one of the most important criteria for leukaemia diagnosis and management. Infant leukaemia is a rare disease that affects children in their first year of life. It has been estimated that approximately one third of infants with acute myeloid leukaemia harbour the t(7;12)(q36;p13) rearrangement in their leukaemic blasts. However, the WHO classification of acute myeloid leukaemia does not yet include the t(7;12) as a separate entity among the different genetic subtypes, although the presence of this chromosomal abnormality has been associated with an extremely poor clinical outcome. Currently, there is no consensus treatment for t(7;12) leukaemia patients. However, with the inferior outcome with the standard induction therapy, stem cell transplantation may offer a better chance for disease control. A better insight into the chromosome biology of this entity might shed some light into the pathogenic mechanisms arising from this chromosomal translocation, that at present are not fully understood. Further work is needed to improve our understanding of the molecular and genetic basis of this disorder. This will hopefully open some grounds for possible tailored treatment for this subset of very young patients with inferior disease outcome. This review aims at highlighting the cytogenetic features that characterise the t(7;12) leukaemias for a better detection of the abnormality in the diagnostic setting. We also review treatment and clinical outcome in the cases reported to date.

## Introduction

**Fig. 1:**
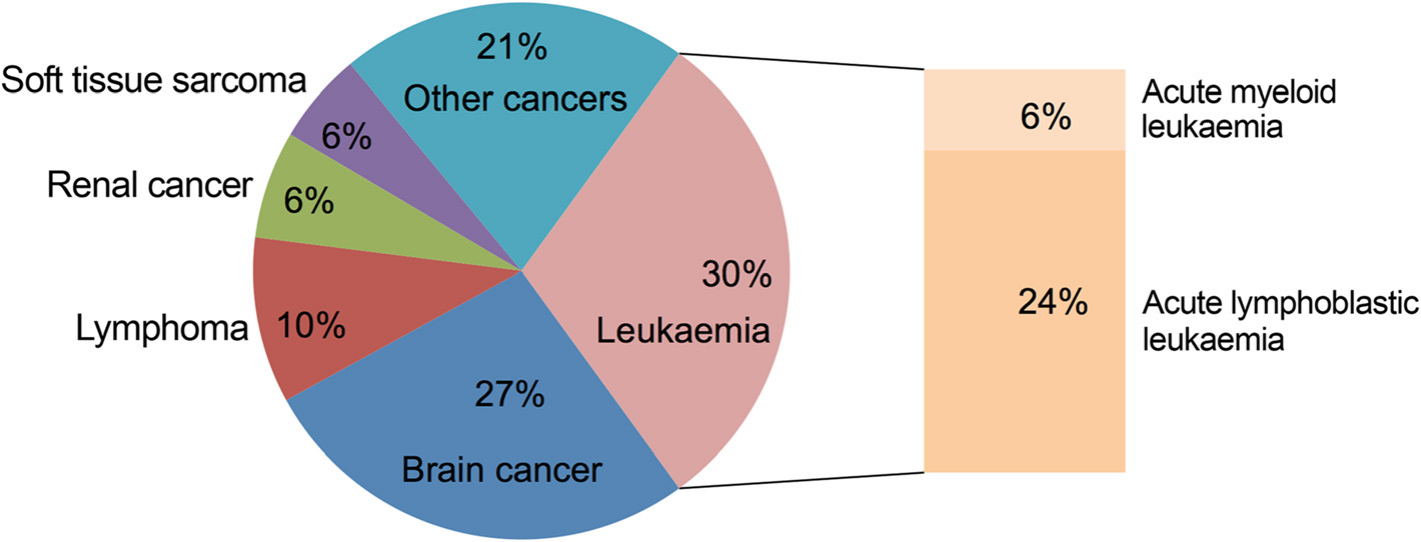
Main types of childhood cancers. The ideogram shows the different proportions of cancers affecting paediatric patients. Acute leukaemia is the most commonly reported cancer in children, with ALL affecting approximately 80 % of patients and AML diagnosed in 15–20 % of patients. Forms of chronic leukaemia and myelodysplastic syndromes are very rare in children and their incidence has been omitted from this graph (based on cancers statistics collected from the National Registry of Childhood Tumours, years 2009–2011, accessed through the Cancer Research UK website)

Leukaemia is the most common type of cancer in childhood (Fig. 1). Among acute leukaemias, one in five is represented by acute myeloid leukaemia (AML), whereas four fifths are acute lymphoblastic leukaemia (ALL). Cancer statistics from the National Registry of Childhood Tumours show a peak at age 2–3 years for the insurgence of ALL, whereas AML is more common within the first year of life and after age 10, with an incidence of 16 cases per million in the United Kingdom. The incidence of AML decreases in children older than 2 years of age, but rises in adolescence when it stays stable until adulthood, reaching its peak in older individuals [1]. Similar statistics have been encountered in the populations of the United States [2, 3].

In the past three decades, there has been a considerable improvement in the cure of childhood leukaemia, reaching a long term survival rate of more than 90 % in ALL and approximately 70 % in AML [3, 4]. However, certain categories of childhood leukaemia are still considered high risk, and this level of risk is dictated by the presence of cytogenetic and molecular genetic markers. The amended world health organization (WHO) leukaemia classification includes the most relevant chromosomal rearrangements to allow proper risk stratification of leukaemia patients [5]. To date, the t(7;12)(q36;p13) rearrangement found in infant AML has not been incorporated in the WHO classification, although it has been associated with poor clinical outcome [3, 6]. This cytogenetic entity has not been associated with a particular morphologic or immunophenotypic subtype [6, 7], but has been found in a range of AML types as well as a case of myelodysplastic syndrome [8]. The scope of this review is to give the reader a comprehensive understanding of the t(7;12) rearrangement at the chromosomal level and the available methods for the detection of this cytogenetic marker for an improved diagnosis and a better estimate of the real incidence of this type of leukaemia. We also review the clinical outcome and the therapeutic approaches that have been adopted in the cases reported to date.

## Review

### The t(7;12) rearrangement: chromosomal appearance and cytogenetic features

**Fig. 2:**
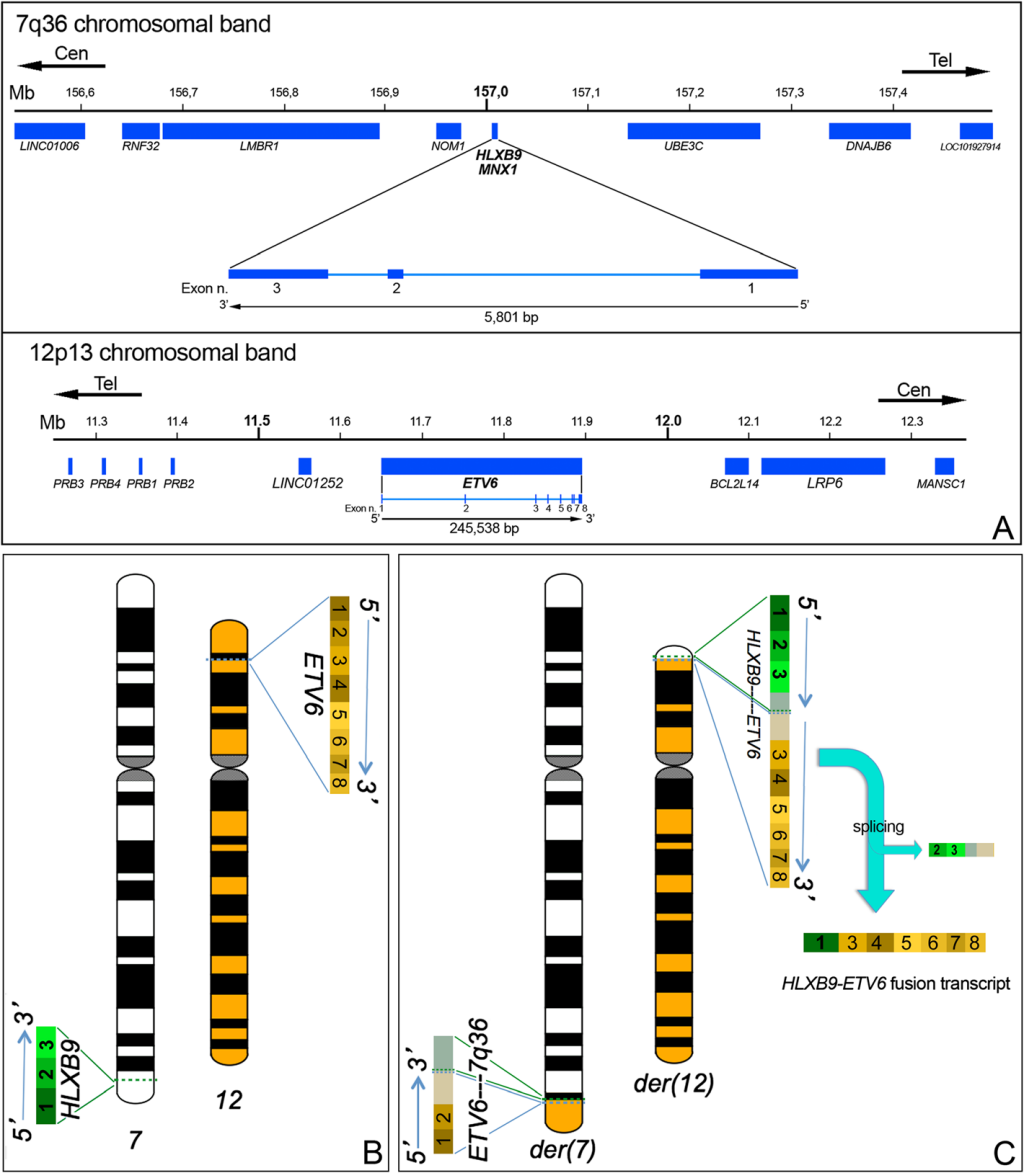
Schematic representation of the t(7;12)(q36;p13) and fusion transcript formation. **a** Representation of the 7q36 and 12p13 regions spanning 1 Mb around the genes of interests. The breakpoints are proximal to the *HLXB9* gene on chromosome 7 and at the 5′ end of the *ETV6* gene on chromosome 12. *HLXB9* is a small gene composed of three exons. *ETV6* is a larger gene composed of eight exons. In both cases the direction of transcription is from the telomeric to the centromeric end. **b** The gene location and direction of transcription of both *HLXB9* and *ETV6* are shown on the ideograms of chromosomes 7 and 12. For each gene all the exons are indicated. **c** Derivative chromosomes 7 and 12: the der(12) harbours the whole *HLXB9* gene and the 3′ portion of *ETV6* including exons 3–8. If a fusion transcript arose from this translocation, splicing of *HLXB9* exons 2 and 3 as well as any genomic material from chromosome 7 downstream to *HLXB9* that was translocated on the der(12) should take place

The t(7;12) rearrangement typically involves the long arm of chromosome 7 at band q36 and the short arm of chromosome 12 at band p13 (Fig. 2). These chromosomal regions reside towards the end of the chromosomes, near the telomeres and involve fragments of similar sizes. The banding patterns of these subtelomeric regions are typically fairly homogeneous and not distinctive of a specific chromosome. For these reasons the t(7;12) rearrangement is a difficult cytogenetic entity to detect microscopically, using conventional methods of chromosome banding. Nevertheless, early reports demonstrate identification of t(7;12) based on banding analysis only [9–11]. Furthermore, the t(7;12) has been found associated with deletions of the long arm of chromosome 7, therefore the der(7) in these cases might be misinterpreted as a del(7q). Fluorescence in situ hybridisation in one del(7)(q22) case, helped revising this abnormality as a der(7)del(7)(q22q36)t(7;12)(q22;p13) [7, 8, 12].

#### Translocation breakpoints

In the t(7;12)(q36;p13), the breakpoints on chromosome 12 are consistently at the 5′ end of *ETV6*, between exons 1 and 3, whereas the breakpoints on chromosome 7 are quite heterogeneous affecting band q36 in regions proximal to the Homeobox HB9 (*HLXB9)* gene [6, 12, 13], also known as *MNX1* (motor neuron and pancreas homeobox 1). There have been descriptions of breakpoints in 7q31 [10], 7q32 [14, 15], and 7q35–36 [8]. However, some of these findings were not validated using fluorescence in situ hybridisation (FISH). Informative FISH probes spanning 7q would have been helpful towards achieving a more accurate definition of the breakpoints in these cases.

#### Cryptic and complex rearrangements

To date, three cases of complex rearrangements harbouring the t(7;12) have been reported [16, 17]. These three way translocations were characterised by FISH and described as (i) t(5;7;12)(q31;q36;p13) [16]; (ii) t(1;7;12)(q25;q36;p13) [16] and (iii) t(7;12;16)(q36;p13;q12) [17] respectively. Due to the breakpoints affecting the terminal regions of both chromosomes 7 and 12, the t(7;12)(q36;p13) is considered a cryptic rearrangement. The use of appropriate FISH probes would surely improve the detection of this abnormality and might uncover a higher proportion of t(7;12) translocations masked by more complex rearrangements, as in the cases of the three way translocations previously described.

#### Additional abnormalities

Interestingly, the majority of t(7;12) cases have been reported in association with specific aneusomies. A recent review of the literature reported that only 2 out of 44 cases harboured the t(7;12) as a sole abnormality [17]. In particular, the presence of one or more additional copies of chromosomes 8, 19 and/or 22 have been recurrently described [6, 7, 15]. The presence of these additional abnormalities has been reported in samples at diagnosis and/or at relapse, indicating that the acquired aneusomies might be sign of clonal evolution and important for the establishment and the survival of the leukaemic clone.

### Molecular mechanisms

#### Is there a fusion gene?

A well-established driver of haematological malignancy when a chromosomal translocation is present, is the formation of an oncogenic fusion gene such as *BCR-ABL* resulting from the t(9;22)(q34; q11) in chronic myeloid leukaemia or *ETV6-RUNX1* in t(12;21)(p13;q22) positive ALL [18]. As a consequence of these gene fusions, a fusion transcript is produced with subsequent generation of a chimeric protein whose altered properties have an impact on the onset of the disease. Reverse transcriptase-polymerase chain reaction (RT-PCR) to identify the fusion transcript in t(7;12) patients showed a fusion of exon 1 of the *HLXB9* gene to either exons 2 or 3 of the *ETV6* gene resulting in two different *HLXB9-ETV6* fusion transcripts: an out of frame longer variant (exon 1 of *HLXB9* to exon 2 of *ETV6*) or an in-frame shorter variant (exon 1 of *HLXB9* to exon 3 of *ETV6*), both fusion transcripts contain the first exon of the *HLXB9* gene as well as the ETS domain (DNA and protein binding domain) and the pointed N terminal domain (PNT) (protein-protein binding domain) of the *ETV6* gene [6, 19–21]. However, the presence of an *HLXB9-ETV6* fusion transcript has been shown only in approximately 50–60 % of t(7;12) patients reported to date (Table 1), whereas the reciprocal *ETV6-HLXB9* transcript has never been observed. This is understandable when looking at the details of the translocation breakpoints at the chromosomal/genomic level. Although the breakpoints on chromosome 12 are well defined and disrupt the *ETV6* gene in a precise location at its 5′ end, on chromosome 7 the breakpoints are scattered in different regions proximal to the *HLXB9* gene. This implies that the whole *HLXB9* gene is transferred onto the der(12), but there is no disruption of the gene itself. Moreover, between *HLXB9* and *ETV6* on the der(12) there should be genomic material of variable extent from 7q, depending on the location of the 7q breakpoint (Fig. 2). This means that an *HLXB9-ETV6* fusion transcript would be generated due to some form of long range splicing. Depending on how efficient this mechanism is, the fusion transcript might fail to be present in all t(7;12) patients. Importantly, there has been no report on the presence of a HLXB9-ETV6 protein to date, therefore the production of a chimeric protein as an oncogenic trigger for the t(7;12) leukaemias is debatable.

**Table 1:** Proportion of patients showing an *HLXB9-ETV6* fusion transcript

No. of t(7;12) patients considered	No. of patients with *HLXB9-ETV6* fusion transcript	Percentage of patients with *HLXB9-ETV6* fusion transcript	Reference
2	2	100	[21]
7	4	57	[6]
2	1	50	[13]
1	1	100	[19]
7	4	57	[20]
6	6	100	[50]

*Notes:* The majority of patients considered in this table were reported as having the t(7;12) in the karyotype of their leukaemic cells. However, in 4 cases [13, 50, 56] the fusion transcript was described in patients with abnormalities of 7q and or 12p, implying that the t(7;12) might have been present, but underestimated in these cases

#### HLXB9 over-expression

As a fusion transcript is detected in only approximately fifty per cent of t(7;12) AML patients, it is debatable that the formation of the fusion gene is the only contributor to leukaemogenesis in these cases. What all the t(7;12) leukaemias have in common is the over-expression of the *HLXB9* gene, suggesting that this, rather than the formation of a fusion gene, might be the driver of leukaemogenesis in these patients. Expression of other genes mapping at 7q36 near the breakpoint has also been evaluated and quantified in t(7;12) patients. However, this analysis showed that expression of *NOM1, LMBR1* and *RNF32* in 7q36 as well as *ETV6* on 12p13 was not different from that observed in AML patients without the t(7;12) and in normal bone marrow [6].

The expression of *HLXB9* in healthy progenitor blood cells in the bone marrow has been investigated by several groups. Early expression studies performed in 1991 showed that bone marrow enriched for CD34-positive cells highly expressed *HLXB9*, whereas unfractionated bone marrow cells expressed *HLXB9* in low levels, and bone marrow cells depleted of CD34-positive cells did not show detectable levels of *HLXB9* [22]. Later on, the same authors reported increased levels of *HLXB9* expression in acute leukaemias that were not seen in the leukaemia patients at remission. These studies suggested a link between *HLXB9* over-expression and leukaemogenesis [23]. Other authors reported a relatively low *HLXB9* expression in normal bone marrow by real time quantitative PCR (RT-Q-PCR) and did not observe *HLXB9* expression in healthy CD34-positive bone marrow cells, but described increased levels in the bone marrow of leukaemia patients with the t(7;12) rearrangement [6, 24]. Observations from our group and by others confirmed elevated *HLXB9* expression in t(7;12) leukaemias as well as in some patients with acute myeloid leukaemia with chromosomal abnormalities other than the t(7;12) [20, 25]. *HLXB9* expression has been associated with hypo-methylation of its promoter in a series of paediatric AMLs. However, in the same study it was found that in childhood ALL the *HLXB9* promoter was hyper-methylated leading to down-regulation of this gene. The authors suggest that *HLXB9* might act as an oncogene in AML, whereas it would act as a tumour suppressor gene in ALL [26]. Over-expression of *HLXB9* has been also reported in lymphoma [27, 28] and cancer types other than haematological malignancies, such as breast cancer [29], testicular cancer [30, 31] and hepatocarcinoma [32]. These studies support the idea that *HLXB9* involvement might be pivotal in other tumours as well as leukaemia. Limited studies have been carried out on the HLXB9 expression at the protein level, whose presence has been demonstrated in bone marrow smear of leukaemia patients with the t(7;12) by immunohistochemistry [16]. Further studies on leukaemia as well as other cancer types are needed in order to clarify whether the presence of elevated *HLXB9* transcript corresponds to proportionate levels of the corresponding protein, or whether translation is regulated by RNA interference pathways [31].

#### HLXB9 over-expression and genome organisation

**Fig. 3:**
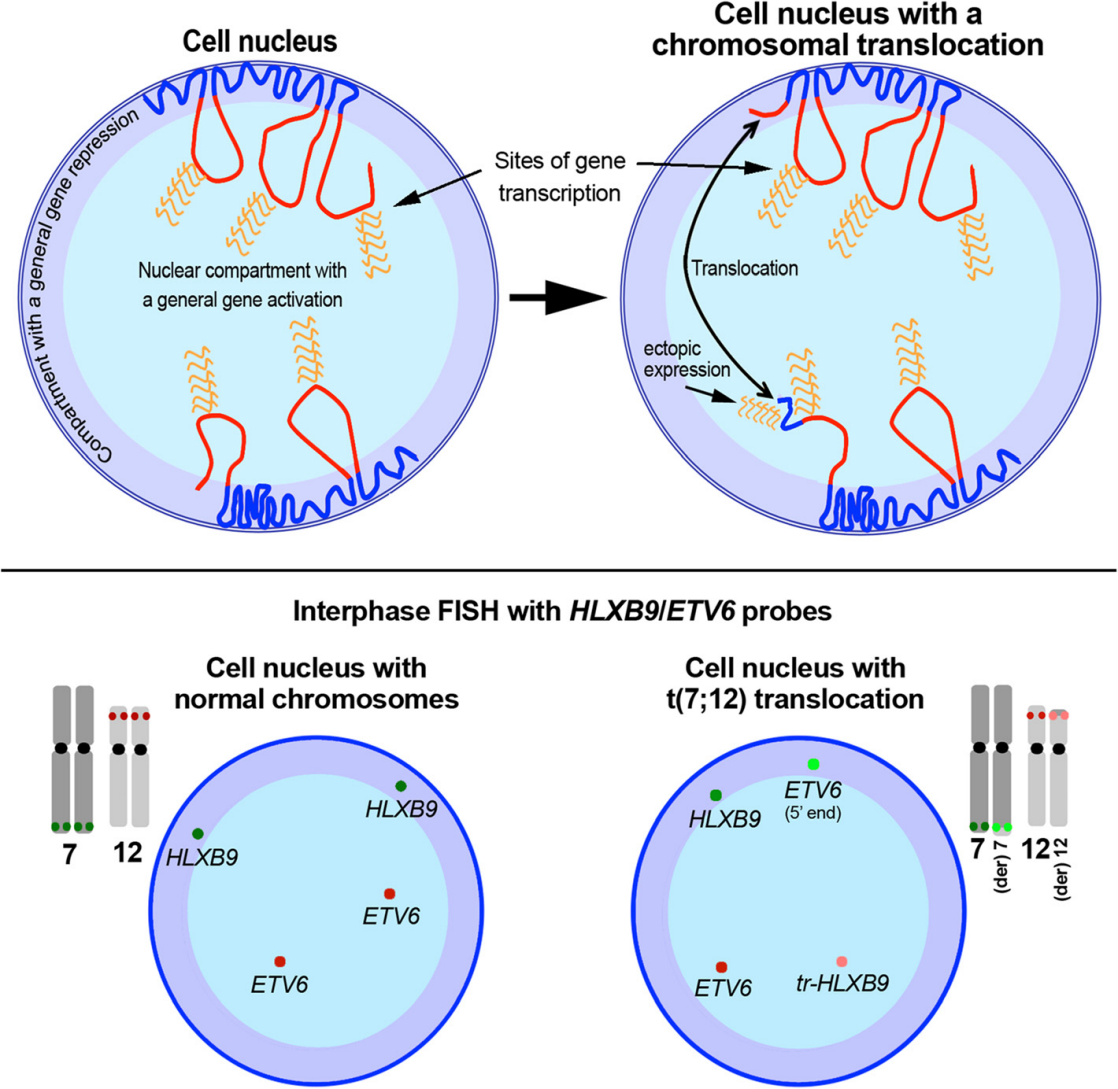
Simplified model of radial chromosome and gene organisation in the cell nuclei. *Upper left*: In the cell nucleus, chromosomes composed of gene dense and gene poor bands are arranged in a zig-zag manner, with the gene poor regions (blue segments) close to the nuclear envelope, in a compact, generally not-transcribed, chromatin organisation. The gene dense regions (red segments) are located in a more internal position, with an open chromatin structure, that favour gene transcription. The interaction of *cis*-acting or *trans*-acting sequences, as well as the presence of specific regulatory factors consents gene activation. *Upper right*: a chromosomal translocation that involves regions normally positioned in different areas of the nucleus could determine an ectopic activation of translocated genes, on the basis of the new nuclear environment where they are repositioned. *Bottom panel*: Schematic representation of the distribution of FISH signals relative to the regions involved in the t(7;12) rearrangement. *Left*: Both copies of *HLXB9* occupy a peripheral positioning, whereas both copies of *ETV6* are localised towards the interior in the nucleus of normal cells. *Right*: The *HLXB9* gene translocated on the der(12) is repositioned towards the nuclear interior, whereas the remaining portion of the *ETV6* gene translocated on the der(7) is repositioned towards the nuclear periphery

An increasing number of studies currently focus on gene expression in the context of the three dimensional (3D) genome organisation in the interphase nucleus [33]. Gene positioning within the different areas of the cell nucleus has been associated with different levels of gene activity, with the general assumption that transcriptionally active genes are localised in the nuclear interior, whereas less active genes tend to be positioned towards the periphery of the nucleus [34–37]. It has also been shown that gene positioning in the nucleus is not fixed, but may change according to different stages of development [38, 39] and in pathologies [40]. It has been demonstrated that the chromosome 3D structure within the interphase nucleus has an influence on the transcriptional activities of specific genes due to a position effect mechanism. For instance, gene expression at a specific locus can be controlled in *cis* by enhancer elements localised at a distance from it, via formation of active chromatin hubs (ACH) [41–43]. Several studies have been conducted to observe the behaviour of cancer loci in the interphase nuclei using different cellular models and some authors proposed that the altered nuclear topography of genes could be used as diagnostic tool in oncology [44, 45]. Studies on cancer fusion genes have shown nuclear repositioning of chromosomes and genes after a translocation event [46–48] and this repositioning has been shown to have an impact on expression profiling [49]. We have observed the behaviour of 7q and 12p loci in the interphase nuclei of leukaemia cells carrying the t(7;12) in a number of patients and shown that the translocation leads to repositioning of the loci of interest (Fig. 3) [20]. What does this imply in terms of gene transcription? We have shown the presence of *HLXB9* transcripts in the same samples with the t(7;12) rearrangement by RT-PCR, however, we did not investigate the origin of the transcript. We assume that the nascent transcript would originate from the der(12), due to the juxtaposition of the *HLXB9* gene to the downstream region of *ETV6.* This would facilitate new interactions between active *cis* elements and the *HLXB9* promoter, leading to the formation of active chromatin hubs and repositioning of the chromatin in the cell nucleus. Alternatively, active *trans* sequences from different chromosomes could activate *HLXB9* by a bridge formation in the new nuclear environment.

#### Gene expression signature of t(7;12) leukaemias and HLXB9 targets

The first study to investigate expression profiling of t(7;12) leukaemias was conducted by Wildenhain et al. [50], who compared leukaemic blasts of patients positive for the presence of the *HLXB9/ETV6* fusion transcript with those with *MLL* rearrangements. It has to be said that these represent the two most common chromosomal rearrangements found in very young AML patients, including infants and mainly children younger than 2 years of age. The authors found that genes expressed in the *MLL* group of patients, were significantly down-regulated in the *HLXB9/ETV6* positive patients. These included HOX genes and genes characteristic for the *MLL*-induced transformation process such as *MEIS1*, *HOXA9* and *C-MYB*. On the other hand, together with over-expression of *HLXB9*, the *HLXB9/ETV6* positive patients showed up-regulation of genes implicated in cell–cell interactions and cell adhesion such as *EDIL3*, *CNTNAP5*, *ANGPT1*, *DSG2*, *ITGA9*, *ITGAV*, *KDR* and *SIGLEC6*. These are known to be implicated in the maintenance of quiescent haematopoietic stem cells (HSC) and homeostasis of the HSC niche. Overall, this study implies that the mechanisms of leukaemogenesis in these two types of childhood leukaemia are fundamentally very different. Evidence would suggest that the *HLXB9/ETV6* positive leukaemias might be initiated through an alterations of interactions between the HSC and the HSC niche. Balgobind et al. [51] used expression microarrays to examine a large cohort of samples from paediatric patients with AML including seven samples from patients with the t(7;12). The authors built a classifier based on 59 genes that enabled them to identify very specific expression profiling signatures predictive of cytogenetic and molecular subtypes. This classification tool, based on the genetic heterogeneity of paediatric AML, is of great diagnostic potential although its feasibility and implementation in the diagnostic setting will have to be further explored. The significance and the impact of *HLXB9* up-regulation in the t(7;12) leukaemias merits further studies. These would shed some light on the pathways triggered by this chromosomal rearrangement. The only report to date to explore possible targets for HB9 (the transcription factor coded by the *HLXB9* gene) in haematopoietic cells was performed on the HL-60 myeloid cell line using ChiP-on-chip and expression profiling analyses [24]. This study describes binding of HB9 to the prostaglandin E receptor 2 (*PGTER2*) promoter resulting in down-regulation of *PGTER2* and consequent reduction of intracellular cAMP level. This down-regulatory effect on *PGTER2* expression was also observed in t(7;12) patients. Together with repression of *PGTER2*, Wildenhain et al. [24], found that HLXB9 expression in the HL60 cell line had mostly a down-regulatory effect on other genes, particularly *ZYX* and *ETS1*. Physiologically, expression of *PGTER2* modulates differentiation of osteoclasts. Up-regulation of *PTGER2* has been shown in immature myeloid precursors. *PTGER2* becomes subsequently down-regulated after the cells have differentiated into osteoclasts [52]. Further work is needed to clarify the implications of *PTGER2* down-regulation on the bone marrow niche and on the differentiation of immature pre-leukaemic cells.

### Diagnostic tools

#### Chromosome banding

The t(7;12)(q36;p13) rearrangement affects the subtelomeric regions of chromosomes 7 and 12 respectively. Due to the small size of the genomic fragments translocated, this rearrangement is difficult to detect using the conventional methods of chromosome banding.

#### Fluorescence in situ hybridisation

**Fig. 4:**
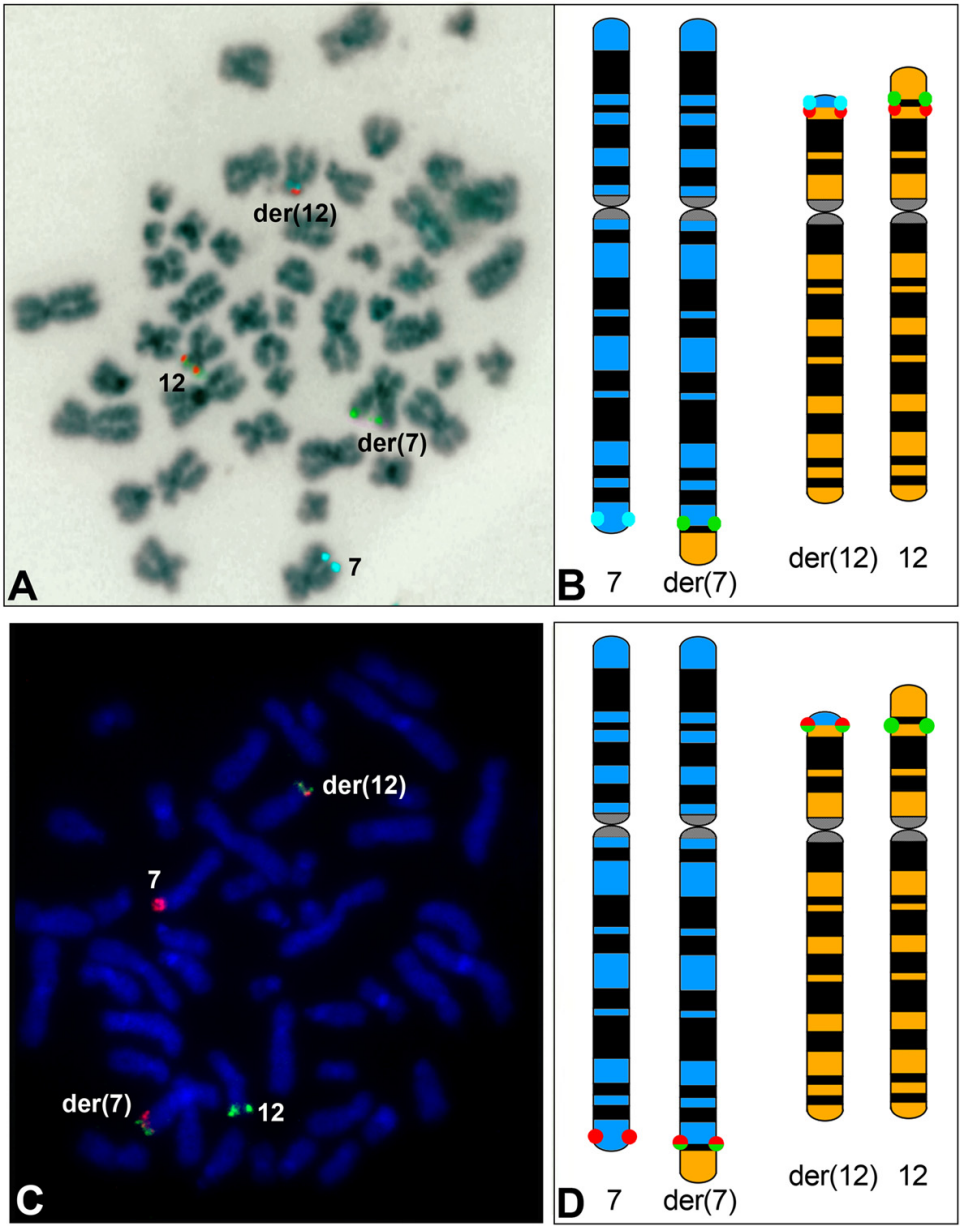
Fluorescence *in situ* hybridisation (FISH) performed on metaphase chromosomes harbouring the t(7;12)(q36;p13). **a** FISH using a three colour approach enables the detection of both normal chromosomes 7 (harbouring only blue hybridisation signals) and 12 (harbouring green and orange fluorescent signals) and their derivatives (green signals on the der(7) and blue and orange signals on the der(12)). The DAPI counterstaining of the chromosomes has been converted into grey scale to simulate a G-banding pattern (figure taken from Naiel et al., 2013 [17]). **b** Schematic representation showing localisation and colour-code of the FISH probes relative to the three colour probe set used. **c** FISH using a two colour approach enables the detection of both normal chromosomes 7 (harbouring only orange hybridisation signals) and 12 (harbouring only green fluorescent signals) and their derivatives carrying orange and green fusion signals. **d** Schematic representation showing localisation and colour-code of the FISH probes relative to the two colour probe set used. It should be noted that the two colour set does not allow to discriminate between the two derivatives based on colour pattern only. The size and morphology of chromosomes 7 and 12 compared to their respective derivatives are very similar, making the identification of the rearrangement difficult without the aid of FISH. Both probe sets have been provided by MetaSystems Gmbh, Altlussheim, Germany

Fluorescence in situ hybridisation (FISH) has been fundamental for the identification of the t(7;12) as a non-random rearrangement [7]. Initially, probes covering the entire *ETV6* gene in conjunction with 7q36 probes have been used for the screening of a relatively large number of paediatric patients. This screening enabled us to estimate the incidence of this translocation of approximately one third of patients with infant leukaemia [7]. It has to be noted that the breakpoints on 7q36 in the t(7;12) patients were found to be heterogeneous and generally proximal to *HLXB9* without disrupting the gene itself [12]. A three colour FISH assay with probes flanking the *HLXB9* gene was developed specifically for the detection of the t(7;12) (q36;p13) (Fig. 4). This assay enabled us to successfully identify cryptic and more complex t(7;12) rearrangements [17]. Commercially available FISH probes for the detection of the t(12;21) translocation are also suitable for the detection of the t(7;12), where a split *ETV6* signal is found on the der(7) and on the der(12). Dual colour whole or partial chromosome painting may also reveal the t(7;12)(q36;p13). However, visualisation of the rearrangement on one or both derivatives might be impaired due to the small size of the translocated fragments.

#### Reverse transcription polymerase chain reaction

Specific primers have been designed for the detection of the *HLXB9/ETV6* fusion at the cDNA level by reverse transcription polymerase chain reaction (RT-PCR) [21]. However, the presence of a fusion transcript by RT-PCR has been identified only in approximately 50 % of t(7,12) leukaemia patients (Table 1). For this reason RT-PCR does not represent a reliable method for the detection of the t(7;12) rearrangements at diagnosis. However, in those patients positive for the *HLXB9/ETV6* fusion transcript, RT-PCR would prove useful for the monitoring of minimal residual disease [53].

#### Expression profiling

Studies based on expression microarrays have shown that the leukaemic cells of patients carrying the t(7;12) have a distinctive expression pattern that allows to discriminate this specific cytogenetic subgroup from other [51]. Microarray based technologies combined with appropriate classifiers constitute potentially a very powerful diagnostic approach, although in a routine diagnostic laboratory the availability of specialised equipment and costs involved might impact on the choice of method.

### Incidence

A literature search has shown that at least 47 leukaemia patients with the t(7;12) have been reported to date, based on the presence of the chromosomal translocation and/or the fusion transcript [17]. To date, there are a very limited number of studies to address the incidence of this translocation, with a quite comprehensive investigation considering a total of 345 paediatric patients [6]. In this study, patients were subdivided in 289 ALL, of which 99 were infants, and 59 AML, of which 18 were infants. It emerged that 5 out of 18 infant AMLs (age 0–12 months) harboured the t(7;12) rearrangement, making the incidence of this rearrangement near one third of infant AML. In addition to the above, the t(7;12) was also found in one infant with ALL and one 18 month old child with AML. A previous study was conducted on a more selected series of leukaemia patients, chosen on the basis of age <20 months and the presence of additional copies of chromosomes 8 and/or 19, as well as abnormalities of 7q and/or 12p [7]. This study showed that 10 out of 23 patients harboured a translocation between 7q and 12p. However, in only 7 cases *ETV6* was involved in the translocation and of these, 6 cases had breakpoint in 7q36 and one in 7q22. Later studies confirmed that the incidence of t(7;12)(q36;p13) was low when considering AML paediatric patients enrolled for clinical trials. These studies reported four t(7;12) cases out of 729 enrolled in the UK MRC AML10 and AML 12; one case out of 454 in AML-BFM 98 and 8 cases out of 981 enrolled in the COG trial AAML0531 [54–56].

### Prognostic significance and treatment options

#### Prognostic significance and clinical outcome

Cytogenetic and molecular markers are important factors to be taken in consideration when stratifying patients into high risk/low risk categories and are of guidance for making treatment choices. The WHO system for the classification of myeloid neoplasms and acute leukemia has been amended to include such information [5]. At present, the t(7;12)(q36;p13) is not included in the WHO classification, although it is recommended that it be considered as a separate entity when assessing infant leukaemia [3]. Review of published data on the clinical outcome of the t(7;12) AMLs reveals inferior outcome with 3 years probabilities of event free survival (EFS) of 0–14 % and overall survival (OS) of 0–28 % (Table 2) [6, 13, 14, 16, 56]. However, one report of a paediatric patient with acute megakaryocytic leukaemia and t(7;12) described achievement of complete remission with chemotherapy alone for 60 months at time of publication [19]. Papers from different clinical trial groups such as the Children’s Oncology Group (COG) [56], UK MRC AML 10 and 12 [53] and the Berlin-Frankfurt-Munich (BFM) [55] included the t(7;12)(q36;p13) in the group of patients with 12p abnormalities and confirmed their association with poor prognosis. It was also stressed the need to consider these patients as a high risk group based on the cytogenetic classification of AML.

**Table 2:** Summary of reports of t(7;12) leukaemias with treatments and clinical outcome

Reference	Study group	No. of patients	CNS status	Treatment/Course/Outcome
[15]	DCLSG/POG	10	5	Treatment N/S
				6 died of leukemia
				1 died of leukemia and infection
				1 died of infection
				1 died of treatment related toxicity
				1 alive, 22 months after BMT in CR1
[13]	CCG	2	2	1 relapsed in bone marrow with bilateral chloromas on the hips. Treated with radiation and BMT, but later died due to disease
				1 relapsed twice, treated with BMT, alive after 2 years
[6]	DCOG	6	N/S	Treatment N/S
				Median EFS: 9 months
				3-year EFS: 0 %
				3-year OS: 0 %
[19]	Japan	1	N/S	Alive after 5 years, treated with chemotherapy only
[16]	Korea	3	N/S	1 received cord blood transplantation at 11 months, relapsed 4 months later, then died at 16 months
				1 died during induction chemotherapy from multiple organ failure
				1 achieved remission, but relapsed at 10 months, and died at 16 months
[56]	COG	8	N/S	Treatment N/S
				3-year EFS: 14 %
				3-year OS: 28 %
				2 of 7 evaluable patients are alive (1 after chemotherapy, 1 after BMT) (personal communication)

*Notes*: *DCLSG* Dutch Childhood Leukemia Study Group; *POG* Pediatric Oncology Group; *CCG* Children’s Cancer Group; *COG* Children Oncology Group; *DCOG* Dutch Childhood Oncology Group; *N/S* not specified; *BMT* bone marrow transplantation; *CR1* first complete remission; *EFS* event-free survival; *OS* overall survival

#### Standard treatment

Standard treatment for paediatric patients with AML includes induction chemotherapy (remission induction) with an anthracycline based regimen which also contains cytarabine. The choice of the anthracycline drug used, as well as the total doses of chemotherapy differ between different centres and collaborative study groups. Most children achieve complete remission with rates of 80–90 % after 2 induction courses and the toxic death rate is in the region of 5 %, with the failures being due to resistant disease [4]. Consolidation or Intensification chemotherapy (post-remission therapy) is essential for cure. It includes the administration of high dose cytarabine possibly in combination with other drugs. Doses, duration, number of drugs as well as scheduling differ between collaborative groups. Currently, treatment related death rates in AML paediatric patients, with the improvement of supportive care and the administration of treatment in experienced units, are now less than 10 % [4].

#### Stem cell transplantation

Haematopoietic stem cell transplantation (HSCT) is considered an effective therapy for treatment of high risk leukaemia. However, the high toxicity which includes a mortality rate reaching up to 20 % in some series, makes it not a standard therapy in the first line treatment of AML patients with favourable cytogenetics, where a high cure rate is achieved with chemotherapy alone. For patients with unfavourable cytogenetics, although the benefit of HSCT is still to be fully explored, HSCT is considered by many groups as the standard of care [57, 58]. When patients relapse, the most important prognostic factors that will determine the outcome of the salvage therapy are the length of first remission and cytogenetics. In the United Kingdom, HSCT is generally used as consolidation therapy as part of salvage treatment with a survival rate of around 40 % achieved irrespective of the source of stem cells. However, it is important to better understand molecular markers and cytogenetics of the disease to identify patients who would most likely benefit from this modality of treatment [59]. To date, although there are no published guidelines about the management of this group, there is increasing interest among paediatric haematologists/study groups to consider intensification of therapy with allogeneic stem cell transplantation in first complete remission [13, 15, 16, 56]. Further study of this important subgroup of infant AML as well as the incorporation of t(7;12) in future risk stratification schemas are needed to better understand the best treatment approach.

## Conclusions

The t(7;12)(q36;p13) is a rare cytogenetic abnormality in paediatric AML and difficult to identify using conventional karyotyping. The use of FISH with appropriate probe sets would enable diagnostic laboratories to identify this cytogenetic entity confidently and to give a better estimate of its occurrence. The incidence of t(7;12) leukaemias is low in overall paediatric AML, but significant in infant leukaemia. There are no published management guidelines for those patients, but it is now acknowledged as a high risk group with poor prognosis and accordingly many are being treated with HSCT in first complete remission. Better understanding of the genetic mechanisms at the basis of t(7;12) leukaemias and the role of *HLXB9* in the establishment of this malignancies would provide grounds for possible tailored therapy.
